# Neurocognitive function in patients with atrial fibrillation undergoing pulmonary vein isolation

**DOI:** 10.3389/fcvm.2022.1000799

**Published:** 2022-11-25

**Authors:** Leon Zwimpfer, Stefanie Aeschbacher, Philipp Krisai, Michael Coslovsky, Anne Springer, Rebecca E. Paladini, Marc Girod, Janik Hufschmid, Sven Knecht, Patrick Badertscher, Jürg H. Beer, Leo H. Bonati, Christine S. Zuern, Laurent Roten, Tobias Reichlin, Christian Sticherling, David Conen, Stefan Osswald, Michael Kühne

**Affiliations:** ^1^Cardiovascular Research Institute Basel, University Hospital Basel, Basel, Switzerland; ^2^Cardiology Division, University Hospital Basel, Basel, Switzerland; ^3^Department of Clinical Research, University Hospital Basel, Basel, Switzerland; ^4^Department of Medicine, Cantonal Hospital of Baden and Molecular Cardiology, University Hospital of Zürich, Zürich, Switzerland; ^5^Reha Rheinfelden, Department of Neurology, University Hospital Basel, University of Basel, Basel, Switzerland; ^6^Department of Neurology and Stroke Center, University Hospital Basel, University of Basel, Basel, Switzerland; ^7^Department of Cardiology, Inselspital, Bern University Hospital, University of Bern, Bern, Switzerland; ^8^Population Health Research Institute, McMaster University, Hamilton, ON, Canada

**Keywords:** neurocognitive function, atrial fibrillation, Montreal Cognitive Assessment, inverse probability of treatment weighting, propensity score, pulmonary vein isolation

## Abstract

**Background:**

Atrial fibrillation (AF) is associated with cognitive dysfunction. However, neurocognitive function in AF patients undergoing pulmonary vein isolation (PVI) has not been well studied. The aim of this analysis is to compare neurocognitive function in patients who did or did not undergo PVI.

**Materials and methods:**

We used data from the Swiss Atrial Fibrillation Cohort study (Swiss-AF), a prospective, observational, multicenter study in Switzerland. Patients with documented AF were enrolled and data of 1,576 patients without history of PVI and with complete information on PVI status and neurocognitive function were used. Information on PVI was collected at baseline and during 1 year of follow-up. Neurocognitive testing was performed at baseline and after 1 year of follow-up, using the Montreal Cognitive Assessment (MoCA), trail making test (TMT) A and B, digit symbol substitution test (DSST) and semantic fluency test (SFT). To investigate the association of PVI with neurocognitive function, we use propensity score matching (1:3) and inverse probability of treatment weighting (IPTW).

**Results:**

The mean age of this population was 74 ± 8 years, 27.1% were women. Overall, 88 (5.5%) patients underwent PVI during 1 year of follow-up. Using ITPW (*n* = 1576), PVI was weakly associated with the MoCA score after adjusting for time since PVI, baseline MoCA score and other covariates (β (95%CI) 1.19 (0.05; 2.32), *p* = 0.04). In the propensity matched comparison (*n* = 352), there was no significant association between PVI and the MoCA score (β (95%CI) 1.04 (−0.19; 2.28), *p* = 0.1). There were no significant associations between PVI and cognitive function when using the TMT A and B, DSST or SFT independent of the method used.

**Conclusion:**

In this population of AF patients, there was no consistent evidence of an association between PVI and neurocognitive function.

**Clinical trial registration:**

[https://clinicaltrials.gov/], identifier [NCT02105844].

## Introduction

Atrial fibrillation (AF) patients face an increased risk of heart failure, stroke, death as well as cognitive impairment, even independent of a history of clinical stroke (1–6). Possible causes for cognitive impairment in AF patients include silent brain infarctions, cerebral hypoperfusion, and high beat-to-beat blood pressure variability due to irregularity of RR intervals (7–9). Pulmonary vein isolation (PVI) is a frequently used and efficient medical intervention to restore sinus rhythm in AF patients with the aim to reduce AF symptoms (10, 11). Despite new evidence showing that PVI reduces heart failure hospitalizations and other cardiac events (12–15), the potential positive or negative effects of rhythm control by means of PVI or the procedure itself on neurocognitive function is unknown. New cerebral lesions after PVI are well described (16–18), and may result in neurocognitive impairment (19). Recent findings indicate cognitive improvement 12 months after successful PVI, mainly in patients with impaired cognitive function prior to PVI (20). In addition, data from a large registry showed that AF patients undergoing PVI had a lower risk of death, stroke and dementia compared to AF patients without PVI after a follow-up time of 3 years (21). Possible explanations for the better cognitive outcome in patients undergoing PVI might be the optimized cardiac output, fewer blood pressure peaks and less variation, and better cerebral perfusion.

The present study aimed to investigate the association between PVI and neurocognitive function in a cohort of patients with AF by using two different statistical approaches.

## Materials and methods

### Study population

The present analysis is based on data from the Swiss Atrial Fibrillation Cohort (Swiss-AF), an ongoing prospective, observational, multicenter cohort study. Overall, 2,415 patients with documented AF were enrolled between 2014 and 2017 across 14 centers in Switzerland. The detailed methodology has been described elsewhere (22). Main inclusion criteria were previously documented AF and age ≥65 years, except for 250 patients aged <65 years that were additionally included to address AF-related absence from work. Patients were excluded if they had short secondary, reversible episodes of AF (e.g., after cardiac surgery), an acute illness within the last 4 weeks or were unable to sign an informed consent (22). The study protocol was approved by the local ethic committees, and written informed consent was obtained from each participant.

**FIGURE 1:**
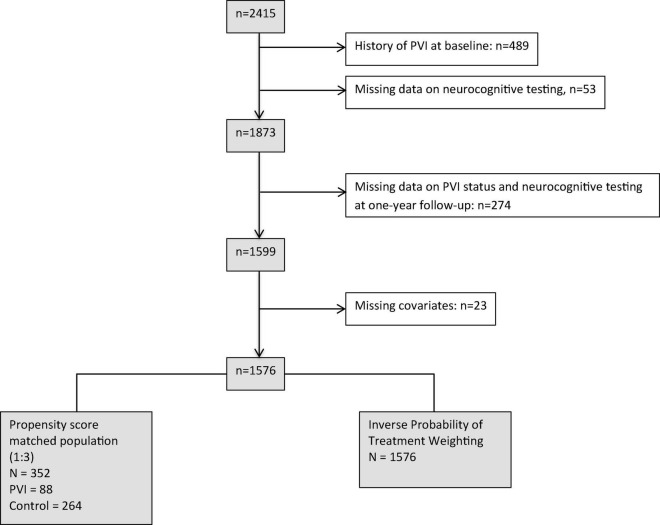
Patient selection.

In this analysis, we aimed to examine the effect of PVI within a 1-year time period on change in neurocognitive function. Consequently, we excluded 839 patients: 542 patients with either a history of PVI prior to enrollment or incomplete neurocognitive data at baseline, and 274 patients due to incomplete neurocognitive tests or missing information on PVI status at follow-up. An additional 23 patients with missing covariates had to be excluded for multivariable adjusted regression analyses, resulting in a dataset of 1,576 patients with complete data. Patient selection is illustrated in Figure 1.

### Study variables

Information on individual patient characteristics, medical history and current medication were collected using standardized case report forms. Body height and weight were measured at baseline, and body mass index (BMI) was calculated by dividing weight in kilograms by height in meters squared. Blood pressure was measured three times in supine position and the mean was used for this analysis. AF type was categorized as paroxysmal versus non-paroxysmal according to the guidelines of the European Society of Cardiology (23). Education level was indicated using standardized questionnaires, where patients were asked to report the highest degree achieved. Educational level was differentiated into basic, middle and advanced educational level. Middle and advanced educational level were combined for this analysis.

### Pulmonary vein isolation

All PVIs were performed between 2014 and 2018, either by radiofrequency ablation (RFA) using an irrigated-tip catheter in conjunction with an electro-anatomic mapping system or a cryoballoon catheter. At baseline and at 1 year of follow-up, we systematically determined whether PVI had been performed. If so, a medical report was requested to confirm the procedure. If a patient had more than one PVI between the baseline visit and 1-year follow-up, the date of the most recent PVI was used. The time between the PVI and the visit date of the follow-up investigation was calculated.

### Neurocognitive testing

Cognitive testing was performed using the Montreal Cognitive Assessment (MoCA) test, the trail making test (TMT, part A and B), the semantic fluency test (SFT), and the digit symbol substitution test (DSST).

In brief, the MoCA test is a frequently used validated global screening assessment for the detection of mild cognitive impairment (24). The total test score ranges from 0 to 30, reflecting cognitive performance in the domains short-term memory, visuospatial capacity (e.g., clock-drawing), language (e.g., animal naming task) and orientation. Furthermore, different facets of executive functions are assessed, including mental flexibility, attention and working memory. In patients with <12 years of education and with a MoCA score of <30 points received an additional point (24). In the Swiss-AF study, a validated German, French and Italian version of the MoCA was used.^1^ Details regarding further cognitive testing using the TMT A and B, the semantic fluency test, and the DSST are provided in the supplement (Supplementary Table 1).

### Statistical analysis

Baseline characteristics were stratified by the presence or absence of PVI between baseline and the 1-year follow-up. Numbers are presented as counts (percentage) for categorical variables, means (±standard deviation) for normally and medians (interquartile range) for highly skewed continuous variables. Groups were compared using a Chi-square test for categorical variables and Student’s *t*-test for continuous outcomes, unless skewed, in which case the Wilcoxon rank-sum test was used. In addition, we report the standardized mean difference (SMD) in baseline characteristics between groups, calculated by difference in mean outcome divided by standard deviation of outcome.

In order to reduce the confounding effect, we pre-specified two different propensity score based methods to investigate the association of PVI with neurocognitive function after the intervention: propensity score matching and inverse probability of treatment weighting (IPTW) (25). First, a propensity score was created by performing a logistic regression analysis using the following variables: age, sex, education (basic vs. middle/advanced), history of heart failure, history of stroke/transient ischemic attack (TIA), history of vascular disease, history of diabetes, history of hypertension, AF type (paroxysmal vs. non-paroxysmal), anticoagulation and/or antiplatelet therapy and history of electrocardioversion (ECV). Due to the assumption of a non-linear association between age and PVI, a data driven restricted cubic spline for age was added to the propensity score model. Based on the propensity score, we created a 1:3 matched *“PVI group”* and *“No PVI group”* using the nearest neighbor method (R package “MatchIT”) (26). To investigate the association between PVI and cognitive function at the follow-up visit we performed multivariable adjusted linear regression analyses to calculate the β-coefficient and the corresponding 95% confidence interval (CI). The β-coefficient reflect the change in neurocognitive score from baseline. The first model was adjusted for baseline cognitive function and time between PVI and follow-up visit. The second model was additionally adjusted for age, sex, education, history of heart failure, history of stroke/TIA, history of hypertension, history of vascular disease, AF-type, history of anticoagulation therapy, and history of ECV.

Second, IPTW was performed in order to investigate the association between PVI and cognition in the whole study population. Patients with a PVI received a weight of 1/propensity score and patients without PVI received a weight of 1/(1-propensity score). Patients with weights >99th quantile (17.76) and those with weights <1st quantile (1.00) were set as the given value. Weights were stabilized by dividing the weight of patients with PVI by the proportion of patients with PVI (0.059) and of patients without PVI by the proportion of patients without PVI (0.941). Multivariable adjusted linear regression analyses were performed as described above (R package “ipw”) (27).

For all analyses, we provide estimates and 95% confidence intervals. We also provide two-sided *p*-values that, in this exploratory analysis, should be interpreted as a continuum indicating how surprising the results would be if the relevant null-hypothesis were true. They should not be taken as confirmatory. We make no correction for multiple testing.

All analyses were performed with R version 3.5.2.

## Results

Baseline characteristics, stratified by the presence or absence of PVI between baseline and 1 year follow-up, are presented in Table 1. Mean age of the study population was 74.3 ± 8 years, 27.1% of all patients were female. Overall, 88 (5.5%) patients underwent PVI during 1 year of follow-up. Median time between PVI and follow-up was 279 (IQR 182–343) days. At baseline, 29 (33%) had AF, 54 (61%) sinus rhythm and 5 (6%) other rhythm. At follow-up, 78 (88.6%) patients had sinus rhythm or an AV-sequential paced rhythm. Compared to patients without PVI, patients with PVI were younger (68 ± 8 vs. 75 ± 8), had a lower CHA_2_DS_2_-Vasc-Score (median: 2.5 (IQR 1–3) vs. 4 (3–5) and less frequently had diabetes (9.1% vs. 17.9%), heart failure (17.0% vs. 27.8%), coronary artery disease (13.6% vs. 34.1%), and a history of stroke or TIA (10.2% vs. 22.7%) (Table 1). With regard to neurocognitive tests, patients with and without PVI had a median MoCA score of 27 vs. 26, a TMT A of 0.65 vs. 0.48, TMT B of 0.24 vs. 0.19, SFT of 21 vs. 18, and a DSST of 51 vs. 42. After propensity score matching (1:3), the baseline characteristics were well distributed between patients with and without a PVI with a SMD < except for diastolic BP (SMD = 0.25), BMI (SMD = 0.16) and paroxysmal AF (SMD = 0.16).

**TABLE 1:** Baseline characteristics of the overall and propensity-score matched population.

	Overall population				Propensity score matched population (1:3)			
	PVI *n* = 88 (5.5)	No PVI *n* = 1488 (94.5)	SMD	*P*-value	PVI *N* = 88	No PVI *N* = 264	SMD	*P*-value
Age, years	68.0 ± 7.7	74.7 ± 7.7	0.88	< 0.001	68.0 ± 7.7	68.7 ± 7.6	0.09	0.45
Female	22 (25.0)	405 (27.2)	0.05	0.74	22 (25.0)	73 (27.7)	0.06	0.73
BMI, kg/m^2^	28.6 ± 4.8	27.8 ± 4.8	0.18	0.10	28.6 ± 4.8	27.8 ± 5.0	0.16	0.21
Current or past smoker	46 (52.3)	818 (55.0)	0.05	0.70	46 (52.3)	149 (56.4)	0.08	0.58
History of diabetes mellitus	8 (9.1)	266 (17.9)	0.26	0.05	8 (9.1)	24 (9.1)	< 0.001	0.99
History of hypertension	61 (69.3)	1075 (72.2)	0.06	0.64	61 (69.3)	179 (67.8)	0.03	0.90
Systolic blood pressure, mmHg	133.7 ± 17.3	134.1 ± 18.6	0.02	0.85	133.7 ± 17.3	133.1 ± 17.7	0.04	0.77
Diastolic blood pressure, mmHg	81.4 ± 12.6	76.9 ± 11.7	0.37	0.001	81.4 ± 12.6	78.4 ± 11.1	0.25	0.04
History of heart failure	15 (17.0)	413 (27.8)	0.26	0.04	15 (17.0)	56 (21.2)	0.02	0.49
History of coronary artery disease	12 (13.6)	507 (34.1)	0.49	< 0.001	12(13.6)	38 (14.4)	0.05	0.99
History of stroke or TIA	9 (10.2)	338 (22.7)	0.34	0.001	9 (10.2)	23 (8.7)	0.05	0.83
CHA_2_DS_2_-VASc score	2.5 (1–3)	4.0 (3–5)	0.85	< 0.001	2.5 (1–3)	2 (2–4)	0.11	0.53
Heart rhythm at baseline			0.39				0.11	0.68
AF	29 (33.0)	750 (50.4)			29 (33.0)	76 (28.8)		
Sinus	54 (61.4)	634 (42.6)			54 (61.4)	168 (63.6)		
Other	5 (5.7)	104 (7.0)			5 (5.7)	20 (7.6)		
Paroxysmal AF	52 (59.1)	619 (41.6)	0.36	0.002	52 (59.1)	135 (51.1)	0.16	0.24
Previous ECV	39 (44.3)	473 (31.8)	0.26	0.002	39 (44.3)	126 (47.7)	0.07	0.67
Antiarrhythmic drug therapy	31 (35.2)	313 (21.0)	0.32	0.02	31 (35.2)	88 (33.3)	0.04	0.85
Oral anticoagulation	81 (92.0)	1344 (90.3)	0.06	0.73	81 (92.0)	243 (92.0)	< 0.001	0.99
Antiplatelet therapy	11 (12.5)	336 (22.6)	0.27	0.04	11 (12.5)	29 (11.0)	0.05	0.85
Education level (middle or high)*	80 (90.9)	1294 (87.0)	0.13	0.36	80 (90.9)	241 (91.3)	0.01	0.99

Values are mean + SD, median (interquartile range) or *n* (%). SMD (standardized mean difference). The *p*-value und SMD compare patients with and without PVI; *p*-values were obtained from Chi-square test for categorical variables and Student’s *t*-test or Wilcoxon rank-sum test for continuous variables. PVI, pulmonary vein isolation; ECV, electrical cardioversion; TIA, transient ischemic attack; CHA_2_DS_2_-VASc, congestive heart failure, hypertension, age ≥ 75 yrs (2 points), diabetes, prior stroke or TIA or thromboembolism (2 points), vascular disease, age 65–74 yrs, female sex. *Basic education: ≤6 yrs; middle: 6 to ≤12 yrs; advanced: ≥12 yrs.

The neurocognitive function scores at baseline and at 1 year of follow-up of the whole population as well as of the propensity score matched sample are presented in Table 2. Differences in the results of the neurocognitive tests between baseline and follow-up were small in the PVI group and in the non-PVI group. Among patients with and without PVI, 53 vs. 48% had a higher MoCA score (*p* = 0.36), and 31 vs. 33% had a lower MoCA score (*p* = 0.68) at 1 year of follow-up.

**TABLE 2:** Results of neuropsychological tests at baseline and follow-up.

		Overall population	Propensity-score matched population	
Cognition test	Visit	(*N* = 1576)	PVI (*n* = 88)	No PVI (*n* = 264)
MoCA	Baseline	26 (23–28)	27 (25–28)	27 (25–28)
	Follow-up	26 (24–28)	27 (26–29)	27 (25–29)
TMT A	Baseline	0.49 (0.37–0.63)	0.65 (0.49–0.78)	0.55 (0.42–0.71)
	Follow-up	0.52 (0.38–0.66)	0.64 (0.50–0.81)	0.61 (0.44–0.74)
TMT B	Baseline	0.19 (0.14–0.26)	0.24 (0.20–0.34)	0.24 (0.17–0.19)
	Follow-up	0.20 (0.13–0.27)	0.28 (0.21–0.32)	0.24 (0.17–0.33)
Semantic fluency test	Baseline	19 (15–22)	20.5 (16–24)	19 (16–24)
	Follow-up	19 (15–23)	20.5 (18–25)	20 (17–24)
DSST	Baseline	42 (34–51)	48 (38–58)	46.5 (38–56)
	Follow-up	42 (34–52)	52 (42–61)	48 (38–58)

Values are medians (interquartile range). MoCA, Montreal Cognitive Assessment; TMT A, trail making test A; TMT B, trail making test B; DSST, digit symbol substitution test.

**FIGURE 2:**
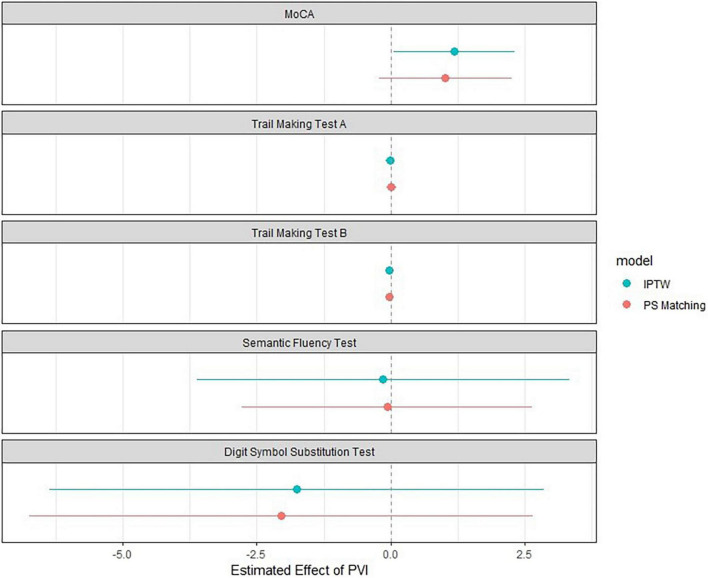
Association between PVI and cognitive function using inverse probability of treatment weighting and propensity score matching. The blue dot represents the effect size using Inverse Probability of Treatment Weighting (*n* = 1576), and the red dot represents the effect size using the propensity-score matched population (*n* = 352). Whiskers correspond to the 95% confidence interval. The model was adjusted for age, sex, corresponding test values at baseline, time since pulmonary vein isolation, history of heart failure, history of stroke or transient ischemic attack, history of hypertension, history of vascular disease, education, atrial fibrillation type, history of anticoagulation, history of electrocardioversion. DSST, digit symbol substitution test; IPTW, inverse probability of treatment weighting; MoCA, Montreal Cognitive Assessment; PVI, pulmonary vein isolation; TMT, trail making test.

Results of the associations between PVI and neurocognitive function are presented in Figure 2 and Tables 3, 4. When using IPTW (Table 3) undergoing PVI was positively associated with the MoCA score with a β-coefficient (95%CI) of 1.35 (0.22; 2.49), *p* = 0.02. In other words, the change in MoCA was 1.35 units higher for patients undergoing PVI than for patients without PVI. The effect size was slightly influenced by the multivariable adjustment, resulting in a smaller effect size and wider confidence intervals [1.19 (0.05; 2.32), *p* = 0.04]. However, the associations of PVI and the other neurocognitive tests were very weak, with very small effect sizes and wide confidence intervals.

**TABLE 3:** Association between PVI and cognitive function using propensity score inverse probability of treatment weighting in patients without history of PVI.

*n* = 1576	Model 1*		Model 2**	
Outcome	β_*PVI*_ (95% CI)	*P*-value	β_*PVI*_ (95% CI)	*P-*value
MoCA	1.35 (0.22; 2.49)	0.02	1.19 (0.05; 2.32)	0.04
TMT A	0.01 (−0.07; 0.10)	0.74	−0.007 (−0.09; 0.08)	0.87
TMT B	−0.03 (−0.07; 0.02)	0.26	−0.03 (−0.08; 0.01)	0.11
Semantic fluency test	0.30 (−3.20; 3.79)	0.87	−0.14 (−3.61; 3.34)	0.94
DSST	−0.52(−5.24; 4.20)	0.83	−1.75 (−6.37; 2.86)	0.46

*Model 1: adjusted for corresponding test value at baseline, time since ablation. **Model 2: adjusted for age, sex, corresponding test value at baseline, time since PVI, history of heart failure, history of stroke/TIA, history of hypertension, history of diabetes, history of vascular disease, education, AF-type, history of anticoagulation, history of ECV. CI, confidence interval; β*_PVI_*, beta coefficient (effect of PVI); MoCA, Montreal Cognitive Assessment; TMT A, trail making test A; TMT B, trail making test B; DSST, digit symbol substitution test.

**TABLE 4:** Association between PVI and cognitive function in the propensity score matched population of patients with atrial fibrillation without a history of a prior PVI.

*n* = 352	Model 1*		Model 2**	
Outcome	β_*PVI*_ (95% CI)	*P*-value	β_*PVI*_ (95% CI)	*P*-value
MoCA	1.04 (−0.19; 2.28)	0.1	1.02 (−0.22; 2.26)	0.11
TMT A	−0.01 (−0.11; 0.09)	0.82	0.01 (−0.09; 0.01)	0.83
TMT B	−0.04 (−0.09; 0.00)	0.07	−0.04 (−0.08; 0.01)	0.12
Semantic fluency test	−0.40 (−3.05; 2.24)	0.76	−0.07 (−2.78; 2.65)	0.96
DSST	−2.41 (−7.09; 2.27)	0.31	−2.05 (−6.76; 2.66)	0.39

*Model 1: adjusted for corresponding test value at baseline, time since ablation. **Model 2: adjusted for age, sex, corresponding test value at baseline, time since PVI, history of heart failure, history of stroke/TIA, history of hypertension, history of diabetes, history of vascular disease, educational status, AF type, oral anticoagulation or antithrombotic treatment, history of electrocardioversion. CI, confidence interval; β*_PVI_*, beta coefficient (Effect of PVI); MoCA, Montreal Cognitive Assessment; TMT A, trail making test A; TMT B, trail making test B; DSST, digit symbol substitution test.

In the propensity score matched population, PVI was not associated with any of the neurocognitive tests (Figure 2, Table 4, and Supplementary Table 3). Multivariable adjustment had no relevant influence on the effect sizes and confidence intervals.

The effect sizes (95% confidence intervals) of the whole regression models are presented in Supplementary Tables 2, 3.

## Discussion

In this analysis we studied the association of PVI within the last year with neurocognitive function in patients with AF. The main new findings of this study are as follows: First, using IPTW, there was a weak positive association between undergoing PVI and global cognitive assessment (MoCA test). However, there was no association between PVI and the MoCA test in a propensity score matched comparison. Based on these results, the evidence of an association is not conclusive. Second, neurocognitive tests (TMT A and B, SFT, DSST) assessing specific neurocognitive functions such as executive functions or psychomotor speed performance (19) speed performance did not show a difference between PVI patients compared to patients not undergoing PVI. Third, AF patients undergoing PVI represented a selected population, were younger and had fewer comorbidities as compared to AF patients not undergoing PVI. However, this was addressed using IPTW and propensity score matching.

Atrial fibrillation has been shown to be associated with cognitive impairment and dementia, a condition with an enormous socio-economic impact (28, 29). However, it is unclear whether cognitive decline in patients with AF can be halted or even improved by PVI, or whether PVI itself may even cause harm with regard to neurocognitive function. There is evidence for asymptomatic cerebral emboli after PVI in a substantial percentage of cases depending on the ablation tool used (4–39%) (18, 30) and adverse short-term effects (after 3 months) on cognition have been reported in a small study (19). A recent case control study by Jin et al. demonstrated improved cognitive function (based on the MoCA test) after 1 year of follow-up in 308 patients undergoing PVI compared to a control group (*n* = 50) (20). Based on our results, we cannot confirm this, even though there was a weak association between PVI and MoCA scores when using IPTW. None of the other associations between PVI and more specific neurocognitive tests were significant, suggesting no conclusive association. Nevertheless, our results may be interpreted as a positive finding, since PVI associated asymptomatic cerebral lesions do not seem to have a detrimental effect on neurocognitive function, at least not after an observation period of 1 year.

To date, the mechanisms underlying the potentially beneficial effect of rhythm control on cognitive function are poorly understood. Improved brain perfusion in sinus rhythm due to better diastolic and systolic function and less beat-to-beat blood pressure variability due to regularity of RR intervals after PVI are possible explanations for the improved cognitive function after PVI (7, 31). From a pathophysiological perspective, better brain perfusion can be assumed in sinus rhythm, independent of the measure used to achieve it. In heart failure patients, PVI was reported to reduce the risk of future heart failure events (13), which in turn may reduce cognitive decline in these patients (32). Whether similar results can be expected in patients who receive medical rhythm control using antiarrhythmic drugs is unclear. The EAST-AFNET 4 study, early rhythm control to standard care showed a similar change in MoCA scores in both groups after 2 years of follow-up. However, the vast majority of patients in EAST-AFNET 4 received early rhythm control by means of antiarrhythmic drug therapy (87%) (15). Whether the potential positive effects of the mentioned mechanisms may be expected after a short to mid-term follow-up is unclear. Long-term studies will be needed to investigate a potential effect of PVI on neurocognitive function.

In our study, different statistical propensity score based approaches were used to investigate the association between PVI and neurocognitive function in order to reduce a potential confounding effect (25). Since no gold-standard method exists in this context, we decided to use two different methods in order to determine whether the findings were consistent. While the results of the propensity score matched population are limited to a smaller and selected population of comparable patients with and without PVI, the results using IPTW are based on the entire population (33). The effect sizes of both statistical approaches are similar. However, the confidence intervals in the selected propensity score matched population are wider and the *p*-value is higher, as expected due to the smaller sample size. This limits the results and does not allow for any definitive conclusions concerning the effect of PVI on cognitive function.

### Strengths and limitations

A strength of this study is the large sample of well-characterized AF patients as well as the comprehensive approach to assess neurocognitive function by using different standardized neuropsychological tests, which capture key aspects of cognitive functioning. Another strength is the application of two different known statistical approaches to investigate the association between PVI and neurocognitive function. Several limitations have to be taken into account when interpreting the results: First, some confounders may have been missed in the choice of variables for the propensity score model and there may still be residual confounding. However, based on this model selection, propensity score matching was successful, resulting in well-balanced strata. Second, a learning effect might have an influence on neurocognitive function testing at follow-up. However, there is no evidence of a differential effect between the two groups. Third, the number of patients with PVI within 1 year seems to be rather low (5.5%). However, it has to be considered that patients who had a PVI before study enrolment were excluded to allow a fair comparison between groups. Nevertheless, this shows that ablation of AF is still performed in selected patients only underscoring the need for matched comparisons using e.g., propensity scores as in this study. Fourth, some results might be non-significant due to the rather small group of patients with a new PVI. Finally, whether our results can be confirmed during long-term follow-up has to be investigated in the future. This is important since AF patients undergoing PVI are generally younger compared AF patients who are not offered PVI.

## Conclusion

Pulmonary vein isolation showed no strong positive or negative effects on most cognitive tests after a follow-up of 1 year. Whether rhythm control using PVI has a long-term effect on neurocognitive function is currently unknown and needs to be investigated in future studies.

## Data availability statement

The datasets presented in this article are not readily available because restrictions by the Ethics Committee. Requests to access the datasets should be directed to MK, michael.kuehne@usb.ch.

## Ethics statement

The studies involving human participants were reviewed and approved by the Ethics Committee Nordwest- und Zentralschweiz, Switzerland and all local Ethics Committees at the study sites. The patients/participants provided their written informed consent to participate in this study.

## Author contributions

LZ, SA, and MK contributed to the conception and design of the study. SA, LZ, PK, AS, RP, MG, and JH organized the clinical database. SA, LZ, and MC performed the statistical analysis. SA and LZ wrote the first draft of the manuscript. SK, PB, JB, LB, CZ, LR, TR, CS, DC, MK, and SO gave intellectual input for manuscript writing. All authors contributed to the article and approved the submitted version.

## Footnotes

1
www.mocatest.org

## Abbreviations

AF: atrial fibrillation
BMI: body mass index
CI: confidence interval
DSST: digit symbol substitution test
ECV: electrocardioversion
IPTW: inverse probability of treatment weighting
MoCA: Montreal Cognitive Assessment
PVI: pulmonary vein isolation
SFT: semantic fluency test
SMD: standard mean difference
Swiss-AF: Swiss Atrial Fibrillation Study
TIA: transient ischemic attack
TMT: trail making test.
