# Supplementary material for: Neurocognitive function in patients with atrial fibrillation undergoing pulmonary vein isolation
Source: Front Cardiovasc Med. 2022 Nov 25;9:1000799. doi: 10.3389/fcvm.2022.1000799 (PMC9732530; doi:10.3389/fcvm.2022.1000799)
Supplement: Supplementary file 1 [file Data_Sheet_1.docx]

**Supplement**

**Neurocognitive Function in Patients with Atrial Fibrillation Undergoing Pulmonary Vein Isolation**

**Swiss-AF investigators**

University Hospital Basel and Basel University: Stefanie Aeschbacher, Katalin Bhend, Steffen Blum, Leo Bonati, David Conen, Ceylan Eken, Urs Fischer, Corinne Girroy, Elisa Hennings, Elena Herber, Vasco Iten, Philipp Krisai, Michael Kühne, Maurin Lampart, Mirko Lischer, Nina Mäder, Christine Meyer-Zürn, Pascal Meyre, Andreas U. Monsch, Luke Mosher, Christian Müller, Stefan Osswald, Rebecca E. Paladini, Anne Springer, Christian Sticherling, Thomas Szucs, Gian Völlmin.

Principal Investigator: Stefan Osswald; Local Principal Investigator: Michael Kühne

University Hospital Bern: Faculty: Drahomir Aujesky, Juerg Fuhrer, Laurent Roten, Simon Jung, Heinrich Mattle; Research fellows: Seraina Netzer, Luise Adam, Carole Elodie Aubert, Martin Feller, Axel Loewe, Elisavet Moutzouri, Claudio Schneider; Study nurses: Tanja Flückiger, Cindy Groen, Lukas Ehrsam, Sven Hellrigl, Alexandra Nuoffer, Damiana Rakovic, Nathalie Schwab, Rylana Wenger, Tu Hanh Zarrabi Saffari. Local Principal Investigator: Nicolas Rodondi, Tobias Reichlin

Stadtspital Triemli Zurich: Christopher Beynon, Roger Dillier, Michèle Deubelbeiss,

Franz Eberli, Christine Franzini, Isabel Juchli, Claudia Liedtke, Samira Murugiah, Jacqueline Nadler, Thayze Obst, Jasmin Roth, Fiona Schlomowitsch, Xiaoye Schneider, Katrin Studerus, Noreen Tynan, Dominik Weishaupt. Local Principal Investigator: Andreas Müller

Kantonspital Baden: Simone Fontana, Corinne Friedli, Silke Kuest, Karin Scheuch, Denise Hischier, Nicole Bonetti, Alexandra Grau, Jonas Villinger, Eva Laube, Philipp Baumgartner, Mark Filipovic, Marcel Frick, Giulia Montrasio, Stefanie Leuenberger, Franziska Rutz. Local Principal Investigator: Jürg-Hans Beer

Cardiocentro Lugano: Angelo Auricchio, Adriana Anesini, Cristina Camporini, Maria Luce Caputo, Francois Regoli, Martina Ronchi. Local Principal Investigator: Giulio Conte

Kantonsspital St. Gallen: Roman Brenner, David Altmann, Michaela Gemperle. Local Principal Investigator: Peter Ammann

Hôpital Cantonal Fribourg: Mathieu Firmann, Sandrine Foucras, Martine Rime. Local Principal Investigator: Daniel Hayoz

Luzerner Kantonsspital: Benjamin Berte, Kathrin Bühler, Virgina Justi, Frauke Kellner-Weldon, Melanie Koch, Brigitta Mehmann, Sonja Meier, Myriam Roth, Andrea Ruckli-Kaeppeli, Ian Russi, Kai Schmidt, Mabelle Young, Melanie Zbinden. Local Principal Investigator: Richard Kobza

Ente Ospedaliero Cantonale Lugano: Elia Rigamonti, Carlo Cereda, Alessandro Cianfoni, Maria Luisa De Perna, Jane Frangi-Kultalahti, Patrizia Assunta Mayer Melchiorre, Anica Pin,Tatiana Terrot, Luisa Vicari. Local Principal Investigator: Giorgio Moschovitis.

University Hospital Geneva: Georg Ehret, Hervé Gallet, Elise Guillermet, Francois Lazeyras, Karl-Olof Lovblad, Patrick Perret, Philippe Tavel, Cheryl Teres. Local Principal Investigator: Dipen Shah

University Hospital Lausanne: Nathalie Lauriers, Marie Méan, Sandrine Salzmann, Jürg Schläpfer. Local Principal Investigator: Alessandra Pia Porretta

Bürgerspital Solothurn: Andrea Grêt, Jan Novak, Sandra Vitelli. Local Principal Investigator: Frank-Peter Stephan

Ente Ospedaliero Cantonale Bellinzona: Jane Frangi-Kultalahti, Augusto Gallino, Luisa Vicari. Local Principal Investigator: Marcello Di Valentino

University of Zurich/University Hospital Zurich: Helena Aebersold, Fabienne Foster, Matthias Schwenkglenks.

Medical Image Analysis Center AG Basel: Jens Würfel (Head), Anna Altermatt, Michael Amann, Marco Düring, Petra Huber, Esther Ruberte, Tim Sinnecker, Vanessa Zuber.

Clinical Trial Unit Basel: Michael Coslovsky (Head), Pascal Benkert, Gilles Dutilh, Milica Markovic, Pia Neuschwander, Patrick Simon, Olivia Wunderlin

Schiller AG Baar: Ramun Schmid

| **Supplementary Table 1:** | | |
| --- | --- | --- |
| **Test** | **Description** | **Scale** |
| The Trail Making Test (TMT) | The Trail Making Test (TMT) is a validated neuropsychological test of executive functioning^1^ usually applied as two parts. In Part A (TMT A), the patient draws lines to connect circled numbers in ascending order (i.e., 1-2-3, etc.) as quickly and accurately as possible, allowing to measure visual attention and psychomotor speed. Similarly, part B (TMT B) requires connecting circled numbers and letters in an alternating numeric and alphabetic order (i.e., 1-A-2-B, etc.), assessing task switching abilities.^2,3^ We measured the time to correctly complete each part in seconds, correspondingly. If the patient was not able to finish the test in 180 seconds (TMT A) or 300 seconds (TMT B), respectively, the test was stopped and the number of correct connections was noted. In order to use data of all patients, we used the number of correct connections per second. | 0-XX correct connections per second |
| Semantic Fluency Test (SFT) | The Semantic Animal Fluency test allows to examining executive abilities and brain functions.[34] Patients have to enumerate as many animal names as possible within 60 seconds. During this task, they need to search for information from semantic memory and to produce the response. Test performance (i.e., total number of word productions) usually depends on the size and location of brain lesions.^4^ While semantic memory and word storage recruit the temporal lobe, modulation of attention and word search depend on processing by the frontal lobe^5^ or the prefrontal-lateral cerebellar system^6^ | 0-XX correct animals in one minute |
| Digit Symbol Substitution Test (DSST) | The DSST is a test of psychomotor speed performance where patients receive a key grid of numbers and matching symbols and a test section with numbers and empty boxes. They have to fill as many empty boxes as possible with the symbol that matches the given number. The score is the number of correct number-symbol matches achieved within 120 seconds, taken to reflect the overall efficiency of cognitive operations.^7^ | 0-XX correct symbols in two minutes |

| **Supplementary Table 2:** Multivariable linear regression models for the association of PVI with change in cognition within one year when using Inverse Probability of Treatment Weighting | | | | | |
| --- | --- | --- | --- | --- | --- |
|  | β coefficient (95% confidence interval) | | | | |
|  | MoCA | TMT A | TMT B | DSST | SF |
| Intercept | 10.9 (8.98; 12.82) | 0.62 (-0.09; 0.08) | 0.23 (0.18; 0.28) | 23.5 (18.3; 28.6) | 14.8 (12.15; 17.50) |
| PVI | 1.19 (0.05; 2.32) | -0.007 (-0.09; 0.08) | -0.04 (-0.08; 0.01) | -1.75 (-6.37; 2.86) | -0.14 (-3.61; 3.34) |
| Baseline cognition | 0.67 (0.62; 0.72) | 0.64 (0.59; 0.69) | 0.73 (0.68; 0.78) | 0.84 (0.81; 0.88) | 0.63 (0.58; 0.68) |
| Time since last PVI | -0.003 (-0.01; 0.00) | 0.000 (0.00; 0.00) | 0.0001 (0.00; 0.00) | 0.009 (-0.01; 0.03) | 0.005 (-0.01; 0.02) |
| Age | -0.04 (-0.05; -0.02) | -0.005 (-0.01; 0.00) | -0.002 (0.00; 0.00) | -0.19 (-0.25; -0.14) | -0.09 (-0.12; -0.06) |
| Female sex | 0.29 (0.01; 0.57) | -0.003 (-0.02; 0.01) | 0.0009 (-0.01; 0.01) | 0.27 (-0.67; 1.20) | 0.13 (-0.34; 0.61) |
| History of heart failure | 0.03 (-0.23; 0.29) | -0.003 (-0.02; 0.01) | -0.008 (-0.02; 0.00) | 0.23 (-0.61; 1.08) | -0.17 (-0.65; 0.32) |
| History of stroke/TIA | -0.34 (-0.65; -0.03) | -0.01 (-0.03; 0.01) | -0.01 (-0.02; -0.01) | -0.81 (-1.76; 0.14) | -0.55 (-1.10; 0.00) |
| History of hypertension | -0.16 (-0.43; 0.10) | 0.007 (-0.01; 0.02) | 0.002 (-0.01; 0.01) | 0.08 (-0.83; 0.98) | -0.28 (-0.76; 0.21) |
| History of diabetes | -0.23 (-0.56; 0.10) | -0.03 (-0.05; -0.01) | -0.008 (-0.02; 0.00) | -1.08 (-2.09; -0.07) | -0.89 (-1.41; -0.37) |
| History of vascular disease | 0.05 (-0.21; 0.31) | -0.007 (-0.02; 0.01) | -0.002 (-0.01; 0.01) | -0.79 (-1.60; 0.02) | 0.31 (-0.17; 0.79) |
| Education higher | 0.77 (0.37; 1.17) | 0.04 (0.02; 0.06) | 0.002 (0.01; 0.03) | 0.82 (-0.28; 1.92) | 0.61 (0.02; 1.21) |
| Paroxysmal AF | -0.07 (-0.31; 0.18) | -0.005 (-0.02; 0.01) | -0.003 (-0.01; 0.00) | 0.36 (-0.44; 1.16) | -0.04 (-0.47; 0.39) |
| Oral anticoagulation | -0.11 (-0.75; 0.52) | -0.02 (-0.06; 0.03) | -0.01 (-0.04; 0.01) | -2.35 (-4.80; 0.10) | -0.95 (-2.20; 0.31) |
| History of electrocardioversion | -0.11 (-0.37; 0.15) | -0.01 (-0.03; 0.00) | 0.002 (-0.01; 0.01) | 0.02 (-0.81; 0.86) | -0.20 (-0.67; 0.27) |

The regression models were adjusted for age, sex, corresponding test value at baseline, time since PVI, history of heart failure, history of stroke/TIA, history of hypertension, history of diabetes, history of vascular disease, education, AF-type, history of anticoagulation, history of ECV

CI= confidence interval, β_PVI_= beta coefficient (Effect of PVI)

MoCA= Montreal Cognitive Assessment. TMT A= Trail Making Test A. TMT B= Trail Making Test B, DSST= Digit Symbol Substitution Test; SF = Semantic Fluency Test

| **Supplementary Table 3:** Multivariable linear regression models for the association of PVI with change in cognition within one year in a propensity score matched population | | | | | |
| --- | --- | --- | --- | --- | --- |
|  | β coefficient (95% confidence interval) | | | | |
|  | MoCA | TMT A | TMT B | DSST | SF |
| Intercept | 12.7 (9.3; 16.0) | 0.47 (0.25; 0.68) | 0.25 (0.16; 0.35) | 20.11 (9.73; 30.49) | 12.06 (6.37; 17.75) |
| PVI | 1.02 (-0.22; 2.26) | -0.01 (-0.09; 0.11) | 0.04 (-0.08; 0.01) | -2.05 (-6.76; 2.66) | -0.07 (-2.78; 2.65) |
| Baseline cognition | 0.61 (0.53; 0.70) | 0.65 (0.57; 0.74) | 0.71 (0.63; 0.79) | 0.86 (0.79; 0.92) | 0.60 (0.51; 0.70) |
| Time since last PVI | -0.002 (-0.01; 0.00) | 0.00 (0.00; 0.00) | 0.00 (0.00; 0.00) | 0.006 (-0.01; 0.02) | 0.004 (-0.01; 0.01) |
| Age | -0.02 (-0.05; 0.01) | -0.03 (-0.01; 0.00) | -0.003 (0.00; 0.00) | -0.12 (-0.24; 0.00) | -0.04 (-0.11; 0.02) |
| Female sex | 0.59 (0.07; 1.11) | -0.04 (-0.08; 0.00) | -0.004 (-0.02; 0.01) | 0.33 (-1.66; 2.31) | -0.12 (-1.27; 1.02) |
| History of heart failure | 0.09 (-0.50; 0.68) | -0.03 (-0.07; 0.02) | -0.007 (-0.03; 0.01) | 1.95 (-0.29; 4.18) | -0.79 (-2.09; 0.50) |
| History of stroke/TIA | -0.04 (-0.88; 0.80) | -0.05 (-0.11; 0.02) | -0.02 (-0.06; 0.01) | -2.33 (-5.51; 0.84) | -1.43 (-3.26; 0.40) |
| History of hypertension | -0.57 (-1.06;-0.08) | 0.007 (-0.03; 0.04) | 0.005 (-0.01; 0.02) | -1.78 (-3.64; 0.09) | -0.52 (-1.59; 0.56) |
| History of diabetes | 0.18 (-0.60; 0.96) | 0.02 (-0.04; 0.08) | -0.006 (-0.03; 0.02) | -0.39 (-3.34; 2.55) | -0.07 (-1.77; 1.62) |
| History of vascular disease | 0.009 (-0.62; 0.64) | -0.02 (-0.06; 0.03) | -0.01 (-0.03; 0.01) | -0.68 (-3.06; 1.70) | 0.42 (-0.96; 1.79) |
| Education higher | 0.91 (0.13; 1.70) | 0.06 (0.00; 0.12) | 0.02 (-0.01; 0.05) | 1.93 (-1.07; 4.94) | 0.68 (-1.04; 2.41) |
| Paroxysmal AF | -0.21 (-0.72; 0.30) | 0.02 (-0.02; 0.05) | -0.00 (-0.02; 0.02) | 0.04 (-1.89; 1.97) | -0.002 (-1.11; 1.11) |
| Oral anticoagulation | -1.01 (-2.05; 0.04) | 0.05 (-0.03; 0.13) | 0.009 (-0.03; 0.05) | -3.90 (-7.86; 0.07) | -0.80 (-3.09; 1.48) |
| History of electrocardioversion | -0.26 (-0.77; 0.25) | -0.02 (-0.06; 0.02) | -0.004 (-0.02; 0.01) | -1.05 (-2.99; 0.88) | -0.22 (-1.33; 0.90) |

The regression models were adjusted for age, sex, corresponding test value at baseline, time since PVI, history of heart failure, history of stroke/TIA, history of hypertension, history of diabetes, history of vascular disease, education, AF-type, history of anticoagulation, history of ECV

CI= confidence interval, β_PVI_= beta coefficient (Effect of PVI)

MoCA= Montreal Cognitive Assessment. TMT A= Trail Making Test A. TMT B= Trail Making Test B, DSST= Digit Symbol Substitution Test; SF = Semantic Fluency Test

**References:**

1. Lezak MD HD, Loring DW, HAnnay HJ and Fischer JS. Neuropsychological assessment. Oxford University Press, New York. 2004;4th edition.

2. Bowie CR and Harvey PD. Administration and interpretation of the Trail Making Test. Nature protocols. 2006;1:2277-81.

3. Arbuthnott K and Frank J. Trail making test, part B as a measure of executive control: validation using a set-switching paradigm. Journal of clinical and experimental neuropsychology. 2000;22:518-28.

4. Lopes M, Brucki SMD, Giampaoli V and Mansur LL. Semantic Verbal Fluency test in dementia: Preliminary retrospective analysis. Dementia & neuropsychologia. 2009;3:315-320.

5. Schweizer TA, Alexander MP, Susan Gillingham BA, Cusimano M and Stuss DT. Lateralized cerebellar contributions to word generation: a phonemic and semantic fluency study. Behavioural neurology. 2010;23:31-7.

6. Baldo JV, Schwartz S, Wilkins D and Dronkers NF. Role of frontal versus temporal cortex in verbal fluency as revealed by voxel-based lesion symptom mapping. Journal of the International Neuropsychological Society : JINS. 2006;12:896-900.

7. Salthouse TA. The processing-speed theory of adult age differences in cognition. Psychological review. 1996;103:403-28.
